# Barriers and enablers for young people, parents and therapists undertaking behavioural activation for depression: A qualitative evaluation within a randomised controlled trial

**DOI:** 10.1111/papt.12452

**Published:** 2023-02-20

**Authors:** Charlotte E. W. Kitchen, Sue Lewis, David Ekers, Lina Gega, Paul A. Tiffin

**Affiliations:** ^1^ Department of Health Sciences University of York York UK; ^2^ School of Health in Social Science University of Edinburgh Edinburgh UK; ^3^ Tees, Esk and Wear Valleys NHS Foundation Trust Durham UK; ^4^ Hull York Medical School University of York York UK

**Keywords:** BA, interviews, mental health, young people

## Abstract

**Background:**

Adolescent depression is common, long‐lasting and debilitating. Behavioural Activation (BA) is a brief, evidence‐based therapy for depression in adults with promising outcomes for young people.

**Objectives:**

We sought to understand how young people, their parents and therapists experienced manualised BA for depression within Child and Adolescent Mental Health Services.

**Design:**

Participants in a randomised controlled trial aged 12 to17 with depression, their parents and therapists were invited to a semi‐structured interview with a researcher to explore their experiences of receiving, supporting or delivering BA.

**Methods:**

Six young people, five parents and five therapists were interviewed. Verbatim interview transcripts were coded using thematic analysis.

**Results:**

Factors that may optimise delivery of BA were: boosting the young person's motivation, tailoring parental input to the young person's needs/wishes and developing a positive collaboration between the young person and therapist. Engagement with treatment may be hindered by a mismatch between BA delivery and young person's preferences, concurrent mental health comorbidities that are not addressed within a wider care package, lack of parental support and therapist preconceptions against manualised therapy or BA.

**Conclusions:**

Manualised BA for young people requires flexibility and adjustment to meet individual and family needs. Therapist preparation could dispel hindering preconceptions about the suitability and potential value of this brief and simple intervention for young people with complex needs and different learning styles.

## INTRODUCTION

Depression is a leading cause of illness and disability in young people worldwide (World Health Organization, 2014). In 10‐ to 19‐year‐olds, the global point prevalence rate for Major Depressive Disorder (MDD) is 8%, with 34% of all adolescents at risk of developing clinical depression (Shorey et al., 2022). Depression in adolescents is characterised by persistent and pervasive sadness, anhedonia, boredom and/or irritability (American Psychiatric Association, 2013; Weisz et al., 2005). Self‐harm and suicide rates are high among adolescents with depression (Patel et al., 2007). Addressing young people's mental health needs and offering access to timely and effective support is crucial for their current and future health and functioning (National Institute for Health and Clinical Excellence [NICE], 2005). Yet many young people with depression do not receive psychological therapy, and although the reasons for this are complex (Radez et al., 2022), one explanation is the limited availability of specialist staff (Department of Health, 2011; Ford et al., 2003). Brief manualised therapies delivered by staff from non‐therapeutic backgrounds or with different levels of experience could enable more widespread access to therapy to meet this need. Behavioural Activation (BA) is one such therapy.

Behavioural Activation is informed by behaviour theory (Kanter et al., 2010), which proposes that when positive reinforcement is lost from a person's environment, depression results and is maintained through a cycle of avoidance of usual activities that, over time, leads to reduced contact with stable sources of positive reinforcement. The introduction of BA aims to break this cycle by increasing time spent in valued, pleasurable or ‘healthy’ behaviours, thereby reintroducing positive reinforcement into the person's life with a view to improving their mood. These activities are introduced or reintroduced through a process called ‘activity scheduling’ which consists of monitoring existing activities and mood, and then collaboratively planning events into the person's everyday routines (Kanter et al., 2010). The brief and simple nature of BA lends itself to easy dissemination to therapists and young people.

In adults, BA is recommended as a first‐line treatment for depression (NICE, 2009) with established clinical and cost‐effectiveness for adults (Ekers et al., 2011; Richards et al., 2016). There is considerably less BA research focusing on young people. There have been three recent systematic reviews of BA for children and young people; one focusing specifically on the treatment of depression (Tindall et al., 2017) and two including BA treatment for a broader range of symptoms (Malik et al., 2021; Martin & Oliver, 2019). Despite the number and quality of the included studies being low and heterogeneity being high, the reviews concluded that BA may be effective in the treatment of depression in children and young people. In recognition of this gap and to generate robust evidence on BA for young people, the UK's National Institute for Health and Care Research (NIHR) has commissioned two large programmes of research to evaluate the clinical and cost‐effectiveness of BA for depression of different levels of severity and across diverse delivery settings (NIHR, 2023).

Only one study has interviewed parents/carers of young people with depression undertaking BA (Dubicka et al., 2022) and very few studies sought feedback from young people who have experienced BA. The largest BA study with adolescents to date did not seek narrative feedback from its participants (McCauley et al., 2016). Smaller, UK‐based studies interviewed young people to explore their experiences of BA in clinical (Dubicka et al., 2022; Shenton et al., 2021; Watson et al., 2021) and educational settings (Arnott et al., 2020; Lewis‐Smith et al., 2021). In UK Child and Adolescent Mental Health Services (CAMHS), qualitative feedback has been limited to studies focusing on anhedonia as a symptom of depression (Watson et al., 2021), low mood without a formal diagnosis of depression (Shenton et al., 2021) or been focused on feedback on intervention materials rather than experiences during BA treatment (Dubicka et al., 2022).

Published studies that explored the experiences and views of clinicians delivering BA were limited in their reporting methods and mode of BA delivery. Tindall et al. (2021) explored the experiences of young people and UK healthcare professionals using a computerised form of BA. Wallis et al. (2012) describe brief qualitative feedback obtained from two social workers trained in BA based in Australian CAMHS, but it is unclear if this information was obtained via survey or interview. Ruggiero et al. (2007) sought feedback during their treatment of a single adolescent in foster care in the United States, although it is unclear how this was achieved or whether it was obtained from the clinician or young person (or both). Cassar et al. (2016) provided reflections from Australian therapists undertaking BA training but did not use a formal methodology.

Already employed internationally, the UK Medical Research Council (MRC) guidelines for the development and evaluation of complex interventions have recently been updated (Skivington et al., 2021). Among the phases of development, the guidelines suggest that young person, parental and therapist experiences can inform the refinement and delivery of BA interventions in a CAMHS setting. Our work is in line with both this latest MRC advice and meets a need identified in Malik et al.'s recent systematic review (Malik et al., 2021) for studies exploring acceptability concerns for BA in young people. Based on qualitative work with young people who received BA, parents supporting their child through treatment and therapists delivering the intervention, we asked two questions of the resulting data on their experiences:

1. What factors may hinder the uptake and completion of BA for young people with depression?
2. How can we facilitate and optimise the delivery of BA for young people with depression?

## METHOD

### Design

This is a qualitative study nested within a Randomised Controlled Trial (RCT) entitled Behavioural Activation for Major Depressive Disorder in Youth, known with the acronym ‘BUDDY’. The trial evaluated the feasibility of BA versus usual care for depression with 22 young people (12–17 years old) in CAMHS. Through the UK National Health Service CAMHS provide help to children and young people experiencing emotional well‐being or mental health difficulties, such as anxiety, depression, self‐harm, eating disorders, obsessive compulsive disorder and psychosis.

An overview of the BUDDY RCT's design, quantitative methods and results has been published elsewhere (Kitchen et al., 2021). In brief, all study participants (young people) were asked to complete standardised questionnaires at three‐months post‐randomisation, and all those in the BA study arm (*n* = 11) were invited to a qualitative interview. Visual comparisons of questionnaire scores on depression, self‐esteem and functioning showed a large shift in a positive direction for the BA group but not for usual care (Kitchen et al., 2021). This manuscript details the qualitative interviews that young people participating in the RCT attended (alongside their parents in all but one case), and the separate interviews participating therapists attended.

The study was approved by a University Research Ethics Sub‐Committee (ref: ESC2/2014/14), the National Research Ethics Committee (ref: 15/NE/0002) and the Trust Research and Development team (ref: 0360/15). The trial was registered with the ISRCTN Registry (ISRCTN52147450).

### Sample

Participants were recruited from three community CAMHS in the North East of England between March 2015 and July 2016. Two sites were in areas in the top 10% and 20% of the *most* deprived neighbourhoods in England, and one was in the *least* deprived 10% (Index of Multiple Deprivation, 2019). Following initial clinical assessment by the CAMHS team, potential participants were directed to the trial either by self‐referral after viewing the study poster, an approach by their CAMHS clinician or by receiving a letter from the research team following a case note review. All families were provided with a recruitment letter and an information sheet (one for parents/carers and an age‐appropriate version for the young person).

Written consent was taken from young people aged 16 to17, if they were able to consent for themselves; for young people under 16, parental consent was necessary along with assent from the young person. Young people aged between 12 and 17 years old with a diagnosis of MDD, confirmed by the Kiddie‐SADS‐Present and Lifetime (K‐SADS‐PL) version (Kaufman et al., 1997), were eligible for trial participation. Qualitative interviews with participants who had received BA treatment (and their parents/carers if the young person agreed) were offered via letter at treatment completion; this was either at three‐month follow‐up or a separate appointment held after their three‐month follow‐up session (if they had not completed BA treatment by the three‐month follow‐up stage). A reminder was also sent via letter if no response was received.

Therapists from across three CAMHS sites where the RCT took place were invited to participate in the interviews. The therapists' experience and roles – defined by their ‘NHS band’ – reflect the diversity of the workforce that routinely offers therapy to young people in the UK's health services, including NHS band 4 (e.g. assistant psychologists), 5 (e.g. low intensity/brief therapies practitioners), 6 (e.g. trainee clinical psychologists) and 7 (e.g. high intensity psychological therapists).

Therapists attended a four‐day training course to deliver an 8‐week manualised therapy (Ekers et al., 2011; McCauley, 2011) based on BA principles (Kanter et al., 2010) and methods refined during a previous study (Arnott et al., 2020). Our BA approach is aligned to the theoretical model by Martell et al. (2001), which focuses on maximising behaviours that act as sources of positive reinforcement while minimising sources of negative reinforcement such as avoidance and procrastination. We did not include a formal assessment of the young people's values as in the BA model developed by Lejuez et al. (2002). Sessions were delivered face‐to‐face, one week apart and lasted around one hour. Therapists delivering BA attended monthly group supervision sessions and had access to individual telephone supervision with the BA trainer as and when they required it. Qualitative interviews with therapists took place during or after administering BA so that staff had a varied caseload of young people at various stages of treatment on which to reflect. All participating therapists were provided with a paper information sheet and consent was sought at the time of the interview.

### Procedure

#### Development of study materials

Members of ‘Youth Speak’, a patient and public involvement group of young people (aged 14–24) were involved as partners in the development of study materials from the outset. Members were instrumental in developing a study name and designing a poster to advertise the trial. Following this input, several Youth Speak members volunteered to be consulted on a more regular, individual basis, which led to alterations to the study documents and procedures. The topic guides for the young person interviews were designed and piloted with these young members. A parent whose children attended CAMHS was recruited via a poster advertisement at one of the recruitment sites. This parent representative was similarly involved in the development, piloting and amendment of the study materials. Therapist topic guides were designed with input from clinical members of the research team.

#### Data collection

Post‐intervention all young people regardless of the number of BA sessions completed (and parents as requested) and therapists in the BA group were invited to attend a qualitative interview with a doctoral researcher. All interviews took place in the therapists' or young person's usual CAMHS site, in a private room. Young people were reimbursed with a £10 voucher. The interviews followed two semi‐structured topic guides (see Supporting Information): one for young people and their parents and one for therapists. Interviewees were encouraged to talk about the topics of most importance to them (Mason, 2017), with open‐ended questions followed by prompts to elicit additional information on views of the trial, the intervention and wider experiences; both positive and negative.

### Data analysis

The purpose of embedding a qualitative component within a RCT was to provide rich, holistic insights into the trial findings from service user, caregiver and therapist perspectives. Randomised controlled trials are rooted in a positivist tradition where the environment is something that can be controlled, resulting in a measurable and singular truth (Barber, 2014). In contrast, qualitative research approaches meaning and knowledge from a constructionist perspective that argues there is no ultimate objective reality, purely a social world that can be perceived by the researcher (Barber, 2014; Mason, 2017). During this qualitative data collection and analysis, this view holds that the researchers and participants are making sense of their reality by attributing and constructing meaning in relation to their experiences. Thus, there is no intention to try to distil this meaning by attempting to quantify experiences.

Qualitative interviews were audio recorded and professionally transcribed verbatim. All participants were assigned an ID number. Transcripts were checked against audio files for accuracy prior to analysis. Transcripts were anonymised to guard against the identification of individuals and places, and pseudonyms substituted to aid the reading of articles and reports. Parents were linked to the pseudonym of their child rather than being assigned their own pseudonyms to provide clarity in linking narratives. An inductive approach to qualitative data analysis was utilised, in which meanings emerged from the data through iterative exploration of the data set, using a thematic analysis approach according to the principles of Braun and Clarke (2006, 2013, 2019, 2021). The analysis focused on remaining true to the participants' voices and developing responses to the research questions. Attention was taken to use quotes in the language participants used, rather than editing to make quotes grammatically correct. This was felt to be particularly important for young people to ensure the presentation of the data was an appropriate representation of their views. Qualitative transcripts were read several times to familiarise researchers with the data. An initial thematic framework was developed across the staff and the young person/parent transcripts and refined by a doctoral researcher and a senior experienced qualitative researcher, based upon the early transcripts from both groups, following which data were assigned to the themes drawn out from the transcripts by the doctoral researcher.

During analysis, the themes, relationship between themes and interpretation were discussed within the research team (doctoral researcher, senior qualitative researcher, Child and Adolescent Psychiatrist, Mental Health Nurse and BA specialist and an expert in Psychotherapies). In addition to the interview data, we used contextual clinical information to assist the analysis, such as diagnosis and whether the participant's depression improved. Rather than being used to corroborate participants' accounts, this information was used as ‘stimulus material’ (Barber, 2014) to situate participant's narratives within the context of therapy delivery by providing information about the number of sessions delivered and therapist perspectives on those sessions. This reflects the acknowledgement of the importance of context in the analysis, as well as content. It also allowed identification of the therapist assigned to each participant, in order to establish links between therapist and patient narratives. Although, data were analysed together to enable corroboration or contradiction between participant's accounts of the phenomena, they were not merged to ensure clarity of the accounts. Crucially, this acknowledges and enables exploration of the sources of apparently alternative explanations.

## RESULTS

### Participating young people, parents and therapists

**FIGURE 1:**
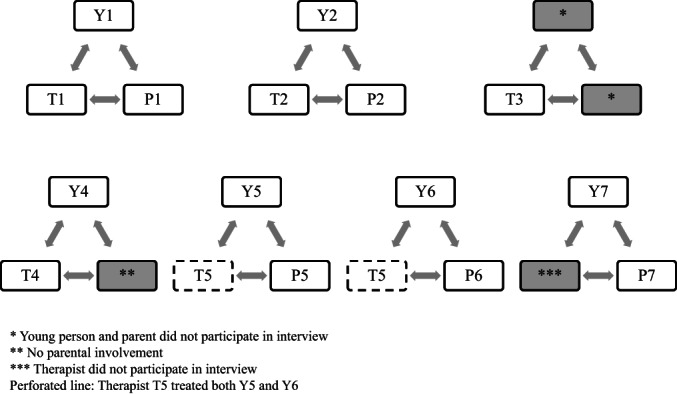
Triads of participating young people (Y), parents (P) and therapists (T).

Six young people, five parents (one parent was unavailable) and five therapists who delivered the intervention participated in the interviews (one declined). Figure 1 below illustrates the relationship between therapists (designated with an ‘T’), young people (designated with an ‘Y’) and parents (designated with a ‘P’). Dyads of young person‐parent have the same number (Y1‐P1, Y2‐P2, etc.), whereas a therapist may have supported more than one family in the trial.

Table 1 provides a summary of participant characteristics from each of the three interviewed groups. Three of the interviewed therapists were male, two were female. All parents interviewed were identified as mothers of the adolescent participants. Most young interviewees were female (one being male), with an age range between 13 and 17 years old (mean 15); half were experiencing mild depression at baseline, the rest moderate or severe. Three months later, half the participants were no longer experiencing MDD, two were experiencing mild depression, one moderate. One young person did not start BA (as they were discharged from the service prior to treatment) so their interview focused on their preconceptions about BA treatment.

**TABLE 1:** Participant characteristics.

Therapist (T)		Young person (Y)					Parent (P)	
Identifier & Pseudonym	Gender	Identifier & Pseudonym	Gender	Age (years)	Depression status at baseline	Depression status at 3 months	Identifier & Pseudonym	Relationship to child
T1 (Geoff)	M	Y1 (Lucy)	F	13	MDD‐mild	No MDD	P1	Mother
T2 (Nicola)	F	Y2 (Estelle)	F	14	MDD‐moderate	No MDD	P2	Mother
T3 (Shane)	M	^a^	–	–	–	–	–	–
T4 (Paul)	M	Y4 (Jessica)	F	17	MDD‐mild + Atypical Depression	No MDD	^b^	–
T5 (Sharon)	F	Y5 (Frankie)	F	17	MDD‐mild	MDD‐mild	P5	Mother
		Y6 (David)	M	17	MDD‐severe + Melancholic Depression (DSM‐IV)	MDD‐moderate	P6	Mother
^c^	–	Y7 (Jennifer)	F	14	MDD‐severe	MDD‐mild	P7	Mother

Abbreviation: MDD, Major Depressive Disorder according to the Kiddie‐SADS‐Present and Lifetime Version.

^a^ Young person (and parents) who were supported by therapist T3 (Shane) did not participate in an interview.

^b^ Parent not present at interview.

^c^ Therapist who supported young person Y7 (Jennifer) did not participate in an interview.

Table 2 provides a summary of the treatment received for those assigned to BA therapy. Number of BA sessions ranged from 2 to 8 (the maximum number offered, with a mean of 5.8 sessions [excluding the young person who did not start treatment]), and duration of sessions ranged from 30 to 75 min.

**TABLE 2:** Participating young peoples' progress with Behavioural Activation (BA).

Young person (Y)	Progress with BA	Number of BA sessions	Duration of BA sessions	Length of BA	Number, duration and purpose of other sessions at 3 months	Number, duration and purpose of other sessions at 3–6 months	Progress within service at 6 months follow‐up
Y1 (Lucy)	Complete	8	1× 45 min 3× 60 min 2× 65 min 1× 70 min 1× 75 min	8 weeks	1× 60 min = Assessment	0	Discharged with no review
Y2 (Estelle)	Complete	8	3× 60 min 3× 45 min 1× 55 min 1× 30 min	11 weeks	1× 50 min = Assessment	0	Discharged. Referred back into the service
Y4 (Jessica)	Non‐start	0	–	0 weeks	0	0	Discharged due to no response to 14‐day letter
Y5 (Frankie)	Incomplete	3	2× 45 min 1× 50 min	6 weeks	1× 50 min = Assessment 1× 45 min = Medication review	1× 45 min unspecified therapy 1× 30 min medication monitoring	Discharged to GP
Y6 (David)	Complete	8	7× 30 min 1× 45 min	13 weeks	1× 60 min = Assessment 5 sessions: 1× 40 min, 1× 35 min, 2× 10 min, 1× unspecified = Medication review	0	No contact with service
Y7 (Jennifer)	Incomplete	2	1× 60 min 1× 55 min	4 weeks	1× unspecified	1× 45 min = Family therapy 1× 10 min = Social worker care coordination 1× 60 min = Cognitive Behavioural Therapy assessment 1× 60 min = Family review 1× (not reported duration) = Cognitive assessment	Discharged due to non‐attendance at two anger management sessions

### Summary of themes and sub‐themes

The thematic analysis of participant responses generated four super‐ordinate themes (*intervention delivery*, *young persons' needs*, *parent involvement* and *therapist effects*) and 13 sub‐themes (as shown in Table 3), which captured the practical and relational factors we could consider in the delivery of BA.

**TABLE 3:** Summary of themes and sub‐themes.

Themes	Sub‐themes	Findings
1. Intervention delivery	1.1. Session frequency	Weekly format endorsed: long enough to complete tasks for next session and short enough to remember previous session. Longer between‐session gaps were perceived as counterproductive
	1.2. Session length	Average session duration was 49 min. Anything shorter was seen as a disadvantage for engagement, especially for a young person with social communication difficulties
	1.3. Number of sessions	Eight sessions sufficient but flexibility is needed; more sessions to address comorbid problems; less sessions to meet limited therapist availability; follow‐up sessions to prevent “cliff‐edge”
	1.4. Therapy manual	Paper‐heavy manual and cluttered sessions. Need lean paper manual or electronic version. Manual not used as “guided self‐help” but as a structure guide for therapist sessions
	1.5. Therapeutic ingredients	The BA model enabled engagement with practical, mood‐lifting activities; goal‐oriented rather than mood‐oriented action
2. Young persons' needs	2.1. Motivation	Motivation, readiness and commitment from young people could aid or hamper progress
	2.2. Concurrent mental health difficulties	Social communication difficulties and mental health conditions other than depression may influence engagement and outcomes
	2.3. Learning style	Young people not grasping the BA model and difficulties remembering information are barriers to engagement
3. Parent involvement	3.1. To involve or not involve parents?	No consensus across families, but agreement within families that the degree and type of parental involvement should match what the young people need and want
	3.2. Benefits of parental involvement	Parents can reinforce and consolidate BA learning; they can provide practical and emotional support to achieve BA goals
	3.3. Downsides to parental involvement	Parents may bring their own problems and anxieties in the sessions; young people may not want to discuss issues in front of parents
4. Therapist effects	4.1. Therapist alliance	Feeling listened to by the therapist was important
	4.2. Therapist equipoise	Preconceptions that BA is not suitable as a standalone treatment, or for ingrained behaviours, or for long‐term mood improvement. BA did not fit with some therapist's established practice. Confidence in delivering it increased “buy‐in”

### Consideration 1: Intervention delivery

#### Session frequency

Therapists, young people and their families favoured regular, weekly BA sessions, as opposed to longer between‐session intervals. One young person and their parent (Y2 Estelle and P2 Estelle's Mother) felt that the weekly format made it easier to remember what had been happening in the young person's life and enabled them to reflect upon the progress of their between‐session goals. Therapists also endorsed a weekly format as being the right balance between having enough time so that ‘…you can make progress. A week is a long time in therapy’ (T2 Nicola), and retaining learning from one session to the next: ‘I think having more than a week between our sessions they remember even less of what we talked about’ (T3 Shane).

Where sessions were not given in the prescribed weekly format, one young person indicated this would have been preferable:

I had [BA sessions] all over the place. Like I would miss a session for three weeks because …[Sharon T5] wasn't there, she was on holiday or something, and I think that sort of affected the entire experience (Y6 David)

David noted these gaps between treatment sessions were to the detriment of his depression treatment: ‘there was long periods of time where I did not have a session, and I think that sort of messed it up’ (David).

#### Session length

Although the sessions were designed to last up to an hour, the average session duration was 49 min (range 30–75 min). Sessions lasting more than 45 min were generally viewed positively. David (Y6) felt that each of his treatment sessions, which all lasted 30 min except one which was 45 min, were too short; he would have preferred each session to last around 50 min. He was not sure whether this might be the case for other young people or whether his Autism Spectrum Disorder (ASD) had an impact upon his ability to communicate with his therapist within these time constraints:

… I find it very hard to speak to people and it's, and when you're sort of just sitting there with somebody it's hard. […] I don't really know how you can sort of explain a lot of things and get across to somebody in half an hour (David)

#### Number of sessions

Most young people felt they had received an appropriate number of BA treatment sessions and would not require more than the eight sessions offered:

I think it was about just right. It was enough time to work on one certain thing and then have another step to go through, and just enough where it got to the point where I could start to help myself a lot more (Y1 Lucy)

For some families, however, the number of treatment sessions offered was not sufficient to address co‐occurring mental health conditions. Estelle's Mother (P2) identified that the number of sessions had been helpful in treating Estelle's (Y2) depression but that she needed more time to focus on her anxiety. Estelle's therapist Nicola (T2) did not feel that BA had been useful in addressing Estelle's generalised anxiety, although it was not clear whether this was due to the content of the BA treatment or the limited number of treatment sessions.

There was mixed feedback from therapists in relation to the optimum amount of treatment sessions. One therapist, Sharon (T5) felt the number of BA sessions was adequate. When reflecting upon the treatment with a complex family (who were not interviewed), another therapist, Paul (T4), reported that it took several sessions to identify and target ingrained avoidance behaviours and so concluded that the flexibility to have more sessions may have been useful. Geoff (T1) indicated that pressure from waiting lists may sometimes require therapists to offer less rather than more sessions, so service capacity was a consideration as much as the needs of young people and their families.

One family also noted the ‘cliff‐edge’ anxiety of transitioning from regular weekly sessions to no support, suggesting that top‐up or follow‐up sessions may be useful to reassure families:

… now it's done… we are pretty much in the dark of what we are going to do now […] It just feels like right that's it, get on with your life now (P2 Estelle's parent)

#### Therapy manual

Therapist feedback suggested there was scope for the manual materials to be further refined. In fact, some therapists and young people felt the length and paper‐heavy nature of the manual acted as a barrier to treatment.

Paul (T4) found the manual needed to be slimmed down so that it was not overwhelming to the therapist, and that the graphics could be improved for the young people (i.e. it was perceived to be ‘a little bit clipart heavy’). Geoff (T1) seconded this sentiment related to the manual graphics, but also commented that the prescriptive format of the manual aided the continuity and management of the therapy delivery, helping the therapist keep up with where they were with the work.

Sharon (T5) felt young people struggled with identifying how they felt at a particular time and in finding the time to record this information on paper (as part of a mood diary). It was suggested an electronic format, such as using a phone app, may be a better way to record this.

#### Therapeutic value

The BA model itself was felt to be important by families and therapists. One therapist revealed that BA ‘…seems to make sense. The whole approach is something I feel young people can grasp rather than some kind of detailed knowledge of psychology or their brain. You know, it's how they are living their life which is causing low mood’ (T4 Paul).

Although Sharon (T5) did not like the BA treatment as a whole, she did like that the BA model offered an explanation of ‘…what has led to somebody feeling the way they did and what maintains them in that state’. Geoff (T1) agreed ‘So I think that certainly from my positive experience it is a model that can be applied to clinical practice and it does have a good outcome’. Echoing this, Shane (T3) said ‘my experience of the BA model was I suppose positive from a clinician point of view’.

Young people were also positive about the BA model. Estelle (Y2) found that BA treatment ‘definitely helped with my low mood’ and felt that during her BA sessions she learnt ‘the right things I needed to learn’. Lucy (Y1) described how ‘it followed the booklet pretty precisely, because obviously it's a test, and it was pretty good talking about things and having the sheets to go back and work on myself. So it wasn't just someone talking and telling me what I could do, it was putting it into practice as well’ highlighting the benefits of a manualised format. Others valued guidance from their therapist: ‘Basically I'd whine for a few minutes about s**t, and then the lady would just actually tell us some good ideas as to how, I like, sort stuff out’ (Y5 Frankie).

All participant groups highlighted the benefits of understanding the links between mood and behaviours, the utility of setting goals and completing between‐session tasks or homework, particularly emphasising the positive role of mood‐lifting, goal‐focused activities. Geoff (T1) felt that the ‘*action orientated*’ focus of the programme was most beneficial. Most therapists and young people highlighted the goal‐setting as a key part of treatment, and valued the progress made throughout this process of breaking larger tasks into smaller ones.

The practical nature of the treatment was viewed as a facilitator. Lucy (Y1) noted ‘I think the most important parts was not just talking about it and what I could do, it's making me put it into practice with the worksheets, and not with anybody else, like, by myself, so I could do it’. Likewise, David (Y6) found BA treatment ‘quite easy to follow’, expressing that the most useful part of treatment was ‘doing the things you'd talk about doing’ such as the between‐session tasks. These tasks comprised of David cooking more meals for himself, for example. Sharon (T5), David's therapist reported his attendance was excellent and ‘he did complete the tasks that were set. Like he arranged and met a friend and went to [a shopping centre]’.

### Consideration 2: Young persons' needs

#### Motivation

For some young people, motivation to participate in BA treatment and/or to improve their depressive symptoms was a key barrier or facilitator in their treatment progress. Estelle (Y2) struggled with low motivation, and initially found it difficult to complete the tasks set by Nicola (T2), ‘…but I would try and put that [the BA] first, and even if I really felt like not doing anything I would still try’. (Estelle).

Therapists also clearly articulated how success in therapy was related to the impetus of each individual: ‘Involvement with particular aspects and particular tasks I think is predominantly down to the young person themselves […]’ (T3 Shane). Nicola (T2) felt the key thing was: ‘…the family have to be motivated to change, not just the child. And there's no point in family being motivated to change if the child is a bit ambivalent…’.

Geoff (T1) seconded the view that the success of treatment was dependent upon the young person's commitment and motivation to change. During the study, he observed that BA treatment worked well when the young person wanted to move forward with treatment, such as in the case of Lucy (Y1), his patient:

I attended all of [the sessions] on the right dates. And I think it was because I knew that I needed help with things, I wanted the help with it. But it had taken quite a bit of time, like two years to come to terms with I would need help with this and I can't do it by myself. And I think some people might miss [the opportunity] if they still can't accept that somebody can help them with it. (Lucy)

#### Concurrent mental health difficulties

Some therapists and young people found the participant's comorbid conditions of anxiety, conduct disorder or ASD, influenced their experiences of BA. For some young people, this meant they required a greater number of BA sessions in order to allow more time to focus on their comorbidity as discussed above; for others, a one‐to‐one ‘talking therapy’ may not have been appropriate. Conversely, other young people who shared these comorbidities (of anxiety and ASD) did not identify them as a barrier to engaging with BA treatment.

Comorbid conditions also interacted with treatment. One young person's diagnosis of an ASD meant they found it difficult to engage with various aspects of their therapy. David (Y6) reflected:

Yeah. I found [BA] somewhat helpful, but I found it quite awkward, because I'm not used to being, I don't really like being in a room with just one person. I find it very hard to speak to people and it's, and when you're sort of just sitting there with somebody it's hard (David)

The one‐to‐one sessions were a social challenge for David, as were many of the activities proposed by his therapist Sharon (T5). David felt that he most likely had a different treatment experience to other young people his age, due to his ASD diagnosis. David's therapist, Sharon found that David's personality and ASD made it difficult for him to be flexible or open‐minded to alternative activities (to his usual solitary interests) during therapy.

#### Learning style

Some therapists questioned whether successful engagement in treatment might be dependent upon intellectual ability:

I don't know if this is about intellectual ability. I found [BA] worked really well with the young person who was intellectually very bright, less well with the one that wasn't, really (T1 Geoff)

…there were some difficulties around understanding the model. And I think from my experience of therapeutic models BA is one of the most simple, which I think is why it's really good for service users. (T3 Shane)

This was evidenced in the ability of young people to complete homework tasks that were set, indicating perhaps that a young person's ability or capacity is an important element in the subsequent success of therapy.

Other therapists and young people suggested that the problem with completing homework tasks between sessions was that they did not remember information from during their session. Two young people reported difficulties remembering the content of sessions or recalling the details of their assigned between‐session tasks, such as completing a mood diary. Jennifer's parent (P7) felt this was due to their child not reading the materials that were provided. Frankie (Y5) found it hard to remember to complete the tasks:

I feel like maybe that mood diary thing that she wanted me to do might have been helpful if I'd actually done it, but I kept forgetting (Frankie)

The young person's parent (P5) challenged this by pointing out that, since treatment finished, Frankie had bought and completed a regular mood diary. The parent thought the mood diary was a good idea as it allowed the young person to revisit past events and this was viewed as an important tool in maintaining a positive mood.

### Consideration 3: Parent involvement in BA

Parents views varied with respect to the optimum level of parental participation in treatment, but, with the exception of one parent/child dyad, each parent and child shared similar views on what worked for them as a family, highlighting the importance of service user choice in therapy.

#### To involve or not involve parents?

One parent (P6) expressed a desire for greater parental involvement in their young person's (Y6 David) BA treatment. Despite valuing the independence that attending his sessions alone brought David, his parent also found that this represented a barrier to providing appropriate parental support during treatment. David's parent suggested that due to his ASD, David only conveyed a limited amount of information about his BA sessions. The parent was left unsure how best to support him during and following his BA treatment.

As was the case for Sharon above, the utility of parental involvement was recognised by other therapists. Therapist interviewees agreed that the level and format of parental involvement had to be determined by considering the young person's preferences. This represented a challenge on occasions where the therapist recognised the need for parental involvement, but the young person did not.

A parent and several therapists suggested the utility of a weekly briefing session for uninvolved parents via telephone to discuss treatment goals. Another family's therapist, Paul (T4) felt the manual materials for parents could be improved. Paul highlighted his work with a young person (who did not attend an interview) and the role her parents played in maintaining her depression and the difficulties of engaging her parents in treatment. Often her parents would drop the young person outside of the CAMHS centre and refuse to come in. Paul felt the parental role in therapy could be tailored to improve the support parents provide to their young person, rather than being focused on more general skills, such as communication. Paul provided the example of this young person's parents thinking it was beneficial for their daughter to spend all night in her bedroom and how they had inadvertently been encouraging low mood behaviour. Paul suggested the family needed to be directly involved in her care, in order for her parents to provide her with appropriate and well‐directed support aimed at improving her depressive symptoms.

Although therapists and one parent felt more parental input would have improved the delivery of BA, there were no young people who suggested they would have preferred more parental involvement. In most cases where parents were involved in young people's BA sessions, young people were content with the amount of parental input and could identify benefits stemming from this collaboration.

#### Perceived benefits of parental involvement

Frankie (Y5) requested that her parent (P5) attend all of her BA sessions. Frankie found this helpful because her parent was able to remind her to employ the skills learnt during treatment in her everyday life. Lucy (Y1) and her family reflected upon the benefits of BA treatment with parental involvement:

I think personally because of what had happened I was quite detached from my family, so at first I didn't want them to be involved. But I think maybe part of it should be getting back the attachment of normal things (Lucy)

I found it better when [Lucy] was talking more with us at home, and I think the way that she was, was improved when that happened more as well (P1 Lucy's parent)

Lucy was comfortable with her parents being involved in her BA treatment but suggested this involvement was most useful towards the end of treatment to help her trouble‐shoot barriers to goal‐setting and activity scheduling.

Some young people and therapists felt parents were a vital source of a support. Therapists recognised the importance of collaborating with parents and considered it to be crucial to treatment success:

Getting the parents on board was really important. […] I can imagine if you did not have a parent that was on board that would be a lot harder. (T2 Nicola)

#### Perceived downsides to parental involvement

Some young people highlighted some potential downsides to parental involvement. The desire of David's parent (P6) to be more involved in his care was not echoed by David (Y6) himself. David's reluctance to have his parent involved was questioned by his parent during the qualitative interview, and it was clear from this exchange that their perspectives differed on what constituted ‘success’ in therapy. Activity scheduling tasks agreed between David and his therapist Sharon (T5) were not valued by his parent. This was illustrated when the parent/child dyad were discussing a recent chance meeting between David and an old friend:

One of the brilliant things that we did on the way back, we bumped into a lovely boy that [David] used to know at school… they've swapped numbers getting off the bus coming here. And they went out, which was brilliant. But [David] has not contacted him since (David's parent)

The parent felt this was an opportunity David had not taken advantage of to connect with an old friend, whereas David retorted:

The thing is… I'd have felt compelled to talk to him, and when we used to hang out we didn't talk a lot. We were just on the computers and we were just sort of, sometimes make like a conversation with each other for like a few seconds and go back on the computers….

This illustrates the difficulties that arise when young people can identify ‘depressed’ or ‘unhealthy’ behaviours, such as sitting on computer games, or unachievable tasks such as meeting a new friend, but their uninvolved parents cannot.

Other young people also raised concerns about the implications of parental involvement. Jessica (Y4) focused upon the stresses related to revisiting past events whilst parents were present, and her concerns about the impact this may have on current relationships within the family. Other families also felt uncomfortable discussing sensitive issues during BA therapy sessions. Estelle's mother felt that her own reticence to be involved stemmed from a feeling that Estelle, and other young people, would be more open with the therapist without a parent being present. Both Estelle and her parent reported being worried about disclosing information during treatment sessions that would be upsetting for each other. This presents a dilemma in terms of how best to involve parents in future iterations of BA manuals.

Geoff (T1) reported variable parental involvement; in one case, he worked with a parent who he felt was very committed (P1 Lucy's parent) whereas in the two other families, the parents were less dedicated in terms of their engagement (neither family attended an interview). Geoff pointed out that although some parents were less actively engaged in their young person's treatment sessions, they remained committed to their young person's recovery, which appeared to be more important than the level of actual involvement.

### Consideration 4: Therapist factors

#### Therapist alliance

Shane (T3) suggested the biggest barrier to successful BA treatment was poor therapeutic alliance between therapist and young person: ‘I think the biggest barrier for service user engagement in BA, in any therapy, in any intervention, in any service, is that therapeutic alliance…’. David (Y6) felt that his therapeutic relationship with Sharon (S5) did not make a difference to his treatment. However, therapist Sharon thought that ‘it might be difficult for him because of his ASD, […] I do think that the most important part of therapy is the relationship […] and BA is not really about the relationship’.

The therapeutic relationship between the young person and their therapist was highlighted as a facilitator for treatment by Frankie's parent (P5) who described their child's (Y5) positive experience: ‘I just think talking to [Sharon T5] really, and having her saying why do not you try this or you are doing really well…’ (Frankie's parent). Both Estelle and her parent (P2) identified having a therapist (T2 Nicola) that they liked impacted upon their treatment experience; ‘We've enjoyed it. Got to know the clinician, did not we, and she was really nice’ (P2 Estelle's parent). Estelle said that ‘she listened’, and that ‘it was nice to have help’. Her parent said ‘…they have got to show empathy have not they, and she did’. Lucy (Y1) valued the comfortable relationship with her therapist Geoff (T1) and Lucy's parent (P1) said ‘I think you felt quite comfortable did not you, talking to [Geoff]…He was very good’. In turn, Geoff felt the therapeutic relationship between the young person and therapist had been strengthened by the weekly format of sessions making it ‘much more positive, much more engaged’, even in cases where treatment was viewed to be less successful.

#### Therapist equipoise

Therapist equipoise is where a therapist holds no preference or knowledge for choosing one treatment over another. This is important to understand when implementing novel therapies because it can impact on implementation and experience during treatment. Some therapists in the trial did not value BA in the same way as other therapies, such as Cognitive Behavioural Therapy. One therapist, Sharon (T5), stated she did not like the BA therapy and would not choose to use it as a stand‐alone treatment. However, she clarified that she found the component BA parts useful and would deliver them alongside other therapeutic approaches.

I think BA is a good little eclectic technique, but I don't really think it's effective on its own unless it's a very simple client that already has lots of resiliencies and resources (Sharon)

This raises questions relating to the impact of how a therapist's belief in the efficacy of an intervention could impact upon its application in clinical services. Understanding why this might be the case is important. Shane (T3) reported that he held a lack of faith that the BA treatment would help the young people he was working with in the long‐term. Instead, Shane chose to rely upon his own clinical judgement in relation to the aspects of the BA treatment he felt would be most helpful to improve their symptoms. Similarly, Nicola (T2) reported:

I had a very amenable young person [Y2 Estelle] and I think that made a big difference. Well she was amenable on the surface… underneath she was very resistant […] [The family were] a very straightforward family to work with on the surface. The difficulties the girl faced were very ingrained though I think for BA

Again, this implies a lack of confidence that the BA treatment would suitably address this young person's symptoms, suggesting there may not be therapist equipoise. Therapists felt there were limitations to a BA approach for depression:

I think Behavioural Activation, I just think it's got those clear limitations around sometimes you do need to go further (T3 Shane)

I find that using, having to use pure BA I feel very constrained and it hampers me. It hampers my intuitiveness and my kind of natural, the natural flow of therapy. (T5 Sharon)

Nicola (T2) reported how her confidence meant she did not struggle with delivery of the treatment; ‘I think in general I quite like chatting to people anyway so for me just part and parcel. Because it's your confidence in yourself is not it, so I did not struggle with delivery’. Similarly, another therapist, Paul (T4), felt a BA manualised approach sat well with his previous background delivering youth work interventions. In contrast, Sharon (T5) felt the prescriptive manual and the acronyms were unhelpful; ‘Well, as I say, I felt constrained and I felt limited and I kept having to interrupt my train of thought…I was more thinking about what I was supposed to do next in a procedural way’.

## DISCUSSION

Young people experiencing depression, their therapists and their parents identified three key factors to best support them during their BA treatment: boosting the young person's motivation, tailoring parental input to the young person's needs and wishes and building a positive working relationship between young people and their therapists. On the contrary, participant experiences of BA suggest that engagement with the intervention could be hindered by five factors: (a) a mismatch between BA delivery and young people's learning styles; (b) concurrent mental health comorbidities that are not addressed within a wider care package; (c) complete lack of parental support; (d) lack of therapist equipoise towards BA as a therapeutic approach and (e) perception of manualised therapy as stifling.

In terms of session frequency, participant feedback was overwhelmingly supportive of the weekly sessions, rather than leaving longer gaps between sessions. Higher session frequency has been associated with greater improvement in mental health outcomes (Cuijpers et al., 2013; Tiemens et al., 2019) and weekly sessions achieve faster improvement than fortnightly sessions (Erekson et al., 2015). Bruijniks et al. (2020) demonstrated that therapy sessions delivered twice rather than once a week can lead to less attrition, quicker response and better outcomes for depression.

There were mixed views from participants in terms of the ideal number of treatment sessions; generally, therapists felt that eight sessions were sufficient to treat depressive symptoms, but some therapists and families in this study were concerned that the treatment did not address comorbid anxiety symptoms or other complex needs. Therapists also felt that delivering the manual content as intended was not feasible during eight hourly sessions. Young people found the 50‐min average session length acceptable. Although the total therapy duration, contact time with a therapist and number of sessions are not associated with treatment effect for depression (Cuijpers et al., 2013), greater flexibility may address therapist and parental concerns, for example by allowing top‐up BA sessions to be offered over a longer period of time following an initial number of BA sessions. This may also prevent the feeling of ‘cliff‐edge’ when therapy ends.

Echoing our findings, Watson et al. (2021) explored young people's experiences of BA and found that motivation was a key factor in the treatment's success. Our study also suggested that this could be enhanced by support from caregivers who are informed about the BA model.

With respect to parental involvement, opinions differed about whether parental attendance during BA sessions was helpful or not. Rather, the preferred approach was to decide on an individual basis for each family. This should be a consideration in recommendations to formalise parental involvement in manualised therapies (Martin & Oliver, 2019). The perceived benefits of parents being present were that knowledge of the BA model enabled them to better judge what support to offer. However, parental involvement risked some parents focusing on their own concerns during the sessions, or their presence inhibiting their young people's willingness to discuss sensitive topics.

Recent work by Shenton et al. (2021) highlighted the importance of the therapeutic relationship in BA, which did not seem to be compromised by the manualised approach in this small study. Our work adds a complementary therapist perspective which demonstrates that client and therapist views and needs with respect to ‘flexibility’ may differ. Manuals need to meet the requirements of young people, their parents and therapists as well as the clinical service, but established practice norms by practitioners may feel counterintuitive to a manualised approach.

Therapists in our study questioned the utility of a standardised approach to treat complex patients because they perceived it to lack agility and collaborative spirit. Flexibility in intervention delivery and adherence would allow for variation in how the BA intervention is delivered and received. Flexibility of delivery was highlighted as a theme in a recent systematic review of the BA literature (Martin & Oliver, 2019) and in a UK CAMHS study of young people undertaking BA (Dubicka et al., 2022). This approach allows implementation of a complex intervention to vary across different contexts yet maintain the integrity of the core intervention components (Skivington et al., 2021). One way to optimise adherence to manualised BA while enabling flexibility for therapists, services and families is to follow a two‐step approach involving *benchmarking* and *adjusting*. First, the therapy manual benchmarks standardised therapy in terms of its frequency, length and duration of sessions, degree of parental involvement and necessary therapeutic ingredients. Second, the delivery of the manual is adjusted to accommodate individual young person requirements, therapist working practices and preferences, service model and capacity and parental availability and ability to be involved. Having a benchmark of what standard therapy should look like, while adjusting its delivery to accommodate individual needs and capabilities, is an expectation in the cultural adaptation of interventions (Hall et al., 2016) and in the reasonable adjustment of care for people with learning and physical disabilities (NICE, 2016). The concept of ‘flexibility within fidelity’ by Kendall et al. (2008) can also be used in therapist supervision to overcome perceptions about the rigidity of manualised therapy. When assessing fidelity to the BA model – or any other intervention – supervisors may need to consider not only whether a therapist adhered to specific therapy principles but also whether they made reasonable adjustments to their delivery of the therapy manual to meet the needs of service users irrespective of their complexity, motivation, external support or learning style.

### Strengths and limitations

Interviews were conducted by a university postdoctoral researcher who was not otherwise involved in the care of the young person, with support from an experienced senior qualitative researcher and clinical team. Our findings highlight the benefits of using a qualitative approach within a RCT context to understand trial outcomes, which is particularly relevant in light of the new development of complex interventions guidance (Skivington et al., 2021). Participants from each therapist–parent–young person triad were interviewed providing a breadth of feedback from the trial. A formal qualitative methodology was used to collect and analyse feedback on BA from both participating young people, parents and therapists, adding considerably to what has previously been an under researched area.

This was a small study based in the North East of England. Not all participating therapists, parents and young people from the BUDDY trial were interviewed; and two of those interviewed who had received BA (*n* = 5) had only two or three sessions, another had none. Staff characteristics, such as previous training or qualifications, were not collected systematically in this study. A fidelity measure was not used in the RCT so the consistency and quality of the BA delivery by individual therapists was managed via standardised training and regular supervision. Given this, it is difficult to identify whether the themes are specific to the particular young person–therapist interaction, rather than the service, or BA more generally. Young people were interviewed alongside their parents, and vice versa, which may have inhibited responses that young people or their parents deemed upsetting. The results should be considered in this context.

### Directions for future research

Future studies should focus on understanding the requirements of CAMHS, individual therapists and families in relation to the level of flexibility needed when undertaking manualised treatments. A particular focus on specific modifications for different groups, such as those with ASD, would be beneficial. Qualitative studies should focus on recruiting those young people who do not start or discontinue BA treatment to further explore their experiences. Therapist training should target preconceptions about the treatment, and research should aim to understand the implications of this on treatment.

## CONCLUSIONS

This study adds to the existing literature on BA and on brief interventions in child and adolescent mental health, specifically focusing on adolescent depression. The qualitative nature of the work enabled a detailed investigation into the barriers and facilitators for a small group of young people, uniquely including both the views of their parents involved in supporting their treatment and their therapists who were delivering it. Our findings emphasised the importance of key, generic elements of psychological therapy when deploying BA in this setting. These included issues related to building and maintaining young people's motivation, therapeutic alliance and taking into account co‐existing mental health conditions when tailoring the treatment delivery. Some more specific issues were also identified. These included the potential benefits of involving parents in the therapy. However, there were suggestions in our data that this should be done sensitively, taking into account the pre‐existing quality of relationships and the stage of treatment, in order to reduce the risk of adverse consequences. We also noted that, despite being a behaviourally‐based, manualised treatment, BA should still be delivered in a flexible way, taking into account the preferences and individual characteristics of both the young person and the wider systemic context. Finally, it seems important to prepare therapists for delivery of BA to young people. This should focus on dispelling hindering preconceptions about the potential value of BA as a standalone therapy which is founded on a behaviourist perspective.

## AUTHOR CONTRIBUTION

CEWK, PAT, SL and DE were responsible for study conception and design. C.E.W.K was responsible for data collection under the supervision of PAT, SL and DE. All authors were responsible for data analysis and drafting the manuscript.

## FUNDING INFORMATION

CEWK was in receipt of an NHS Doctoral Studentship (2013–2017) during the data collection for this project and is currently funded through an Economic and Social Research Council Fellowship; the support of these funders is gratefully acknowledged.

## CONFLICT OF INTEREST STATEMENT

Co‐authors LG, DE and PAT hold grants from the National Institute for Health Research for research projects relevant to this paper's intervention and population.

## Supporting information


Appendix S1.

## Data Availability

Data available on request due to privacy/ethical restrictions.

## References

[papt12452-bib-0001] American Psychiatric Association (Ed.). (2013). Diagnostic and statistical manual of mental disorders (5th ed.). American Psychiatric Association.

[papt12452-bib-0002] Arnott, B. , Kitchen, C. E. W. , Ekers, D. , Gega, L. , & Tiffin, P. A. (2020). Behavioural activation for overweight and obese adolescents with low mood delivered in a community setting: Feasibility study. BMJ Paediatrics Open, 4, e000624. 32399504 10.1136/bmjpo-2019-000624 PMC7204816

[papt12452-bib-0003] Barber, R. (2014). Introducing qualitative research: A student's guide. SAGE Publications.

[papt12452-bib-0004] Braun, V. , & Clarke, V. (2006). Using thematic analysis in psychology. Qualitative Research in Psychology, 3(2), 77–101.

[papt12452-bib-0005] Braun, V. , & Clarke, V. (2013). Successful qualitative research: A practical guide for beginners. Sage.

[papt12452-bib-0006] Braun, V. , & Clarke, V. (2019). Reflecting on reflexive thematic analysis. Qualitative Research in Sport, Exercise and Health, 11(4), 589–597.

[papt12452-bib-0007] Braun, V. , & Clarke, V. (2021). One size fits all? What counts as quality practice in (reflexive) thematic analysis? Qualitative Research in Psychology, 3(18), 328–352.

[papt12452-bib-0008] Bruijniks, S. J. E. , Lemmens, L. H. J. M. , Hollon, S. D. , Peeters, F. P. M. L. , Cuijpers, P. , Arntz, A. , Dingemanse, P. , Willems, L. , Van Oppen, P. , Twisk, J. W. R. , Van den Boogaard, M. , Spijker, J. , Bosmans, J. , & Huibers, M. J. H. (2020). The effects of once‐ versus twice‐weekly sessions on psychotherapy outcomes in depressed patients. The British Journal of Psychiatry, 216(4), 222–230. 32029012 10.1192/bjp.2019.265

[papt12452-bib-0009] Cassar, J. , Ross, J. , Dahne, J. , Ewer, P. , Teesson, M. , Hopko, D. , & Lejuez, C. W. (2016). Therapist tips for the brief behavioural activation therapy for depression ‐ revised (BATD‐R) treatment manual practical wisdom and clinical nuance. Clinical Psychologist, 20(1), 46–53. 29720886 10.1111/cp.12085 PMC5926241

[papt12452-bib-0010] Cuijpers, P. , Huibers, M. , Ebert, D. D. , Koole, S. L. , & Andersson, G. (2013). How much psychotherapy is needed to treat depression? A meta‐regression analysis. Journal of Affective Disorders, 149(1–3), 1–13. 23528438 10.1016/j.jad.2013.02.030

[papt12452-bib-0011] Department of Health . (2011). Talking therapies: A four‐year plan of action. Department of Health.

[papt12452-bib-0012] Dubicka, B. , Marwedel, S. , Banares, S. , McCulloch, A. , Tahoun, T. , Hearn, J. , & Kroll, L. (2022). Feasibility study of a new behavioural activation programme for young people with depressed mood. Child and Adolescent Mental Health, 27(2), 131–137. 34028154 10.1111/camh.12474

[papt12452-bib-0013] Ekers, D. , Godfrey, C. , Gilbody, S. , Parrott, S. , Richards, D. A. , Hammond, D. , & Hayes, A. (2011). Cost utility of behavioural activation delivered by the non‐specialist. The British Journal of Psychiatry, 199(6), 510–511. 21947655 10.1192/bjp.bp.110.090266

[papt12452-bib-0014] Erekson, D. M. , Lambert, M. J. , & Eggett, D. L. (2015). The relationship between session frequency and psychotherapy outcome in a naturalistic setting. Journal of Consulting and Clinical Psychology, 83(6), 1097–1107. 26436645 10.1037/a0039774

[papt12452-bib-0015] Ford, T. , Goodman, R. , & Meltzer, H. (2003). Service use over 18 months among a nationally representative sample of British children with a psychiatric disorder. Clinical Child Psychology and Psychiatry, 8, 37–51.

[papt12452-bib-0016] Hall, G. C. , Ibaraki, A. Y. , Huang, E. R. , Marti, C. N. , & Stice, E. (2016). A meta‐analysis of cultural adaptations of psychological interventions. Behavior Therapy, 47(6), 993–1014. 27993346 10.1016/j.beth.2016.09.005

[papt12452-bib-0017] Index of Multiple Deprivation . (2019). Indices of Deprivation 2019 explorer . http://dclgapps.communities.gov.uk/imd/iod_index.html

[papt12452-bib-0018] Kanter, K. W. , Manos, R. C. , Bowe, W. M. , Baruch, D. E. , Busch, A. M. , & Rusch, L. C. (2010). What is behavioral activation? A review of the empirical literature. Clinical Psychology Review, 30, 608–620. 20677369 10.1016/j.cpr.2010.04.001

[papt12452-bib-0019] Kaufman, J. , Birmaher, B. , Brent, D. , Rao, U. , Flynn, C. , Moreci, P. , Williamson, D. , & Ryan, N. (1997). Schedule for affective disorders and schizophrenia for school‐age children‐present and lifetime version (K‐SADS‐PL): Initial reliability and validity data. Journal of the American Academy of Child and Adolescent Psychiatry, 36(7), 980–988. 9204677 10.1097/00004583-199707000-00021

[papt12452-bib-0020] Kendall, P. C. , Gosch, E. , Furr, J. M. , & Sood, E. (2008). Flexibility within fidelity. Journal of the American Academy of Child & Adolescent Psychiatry, 47(9), 987–993. 18714195 10.1097/CHI.0b013e31817eed2f

[papt12452-bib-0021] Kitchen, C. E. W. , Tiffin, P. A. , Lewis, S. , Gega, L. , & Ekers, D. (2021). Innovations in Practice: A randomised controlled feasibility trial of Behavioural Activation as a treatment for young people with depression. Child and Adolescent Mental Health, 26(3), 290–295. 32725758 10.1111/camh.12415

[papt12452-bib-0022] Lejuez, C. W. , Hopko, D. R. , & Hopko, S. D. (2002). The brief behavioral activation treatment for depression (BATD): A comprehensive patient guide. Pearson Custom Publishing.

[papt12452-bib-0023] Lewis‐Smith, I. , Pass, L. , Jones, D. J. W. , & Reynolds, S. (2021). “…if I care about stuff, then other people care about me”. Adolescents' experiences of helpful and unhelpful aspects of brief behavioural activation therapy for depression. Psychotherapy Research, 31(8), 1067–1078. 33710945 10.1080/10503307.2021.1898692

[papt12452-bib-0024] Malik, K. , Ibrahim, M. , Bernstein, A. , Kodihalli Venkatesh, R. , Rai, T. , Chorpita, B. , & Patel, V. (2021). Behavioral activation as an ‘active ingredient’ of interventions addressing depression and anxiety among young people: A systematic review and evidence synthesis. BMC Psychology, 9, 150. 34615559 10.1186/s40359-021-00655-x PMC8494510

[papt12452-bib-0025] Martell, C. R. , Addis, M. E. , & Jacobson, N. S. (2001). Depression in context: Strategies for guided action. W W Norton & Co.

[papt12452-bib-0026] Martin, F. , & Oliver, T. (2019). Behavioral activation for children and adolescents: A systematic review of progress and promise. European Child & Adolescent Psychiatry, 28(4), 427–441. 29476253 10.1007/s00787-018-1126-z PMC6445819

[papt12452-bib-0027] Mason, J. (2017). Qualitative researching. SAGE Publications Ltd.

[papt12452-bib-0028] McCauley, E. , Gudmundsen, G. , Schloredt, K. , Martell, C. , Rhew, I. , Hubley, S. , & Dimidjian, S. (2016). The adolescent behavioral activation program: Adapting behavioral activation as a treatment for depression in adolescence. Journal of Clinical Child and Adolescent Psychology, 45(3), 291–304. 25602170 10.1080/15374416.2014.979933 PMC6107350

[papt12452-bib-0029] McCauley, E. A. (2011). *Behavioural Activation Manual* (Personal correspondence).

[papt12452-bib-0030] National Institute for Health and Care Excellence . (2016). *Mental health problems in people with learning disabilities: Prevention, assessment and management* (NICE guideline NG54, recommendations 1.9.1–1.9.4, 1.9.8 and 1.9.9). 27683921

[papt12452-bib-0031] National Institute for Health and Care Research . (2023). Funding Awards . https://fundingawards.nihr.ac.uk/award/NIHR201174, https://fundingawards.nihr.ac.uk/award/NIHR132808

[papt12452-bib-0032] National Institute for Health and Clinical Excellence . (2005). *Depression in children and young people: Identification and management in primary, community and secondary care* (National Clinical Practice Guideline No. 28).

[papt12452-bib-0033] National Institute for Health and Clinical Excellence . (2009). *Depression: The treatment and management of depression in adults* (Partial update of Clinical Practice Guideline No. 23).

[papt12452-bib-0034] Patel, V. , Flisher, A. J. , Hetrick, S. , & McGorry, P. (2007). Mental health of young people: A global public‐health challenge. Lancet, 369, 1302–1313. 17434406 10.1016/S0140-6736(07)60368-7

[papt12452-bib-0035] Radez, J. , Reardon, T. , Creswell, C. , Orchard, F. , & Waite, P. (2022). Adolescents' perceived barriers and facilitators to seeking and accessing professional help for anxiety and depressive disorders: A qualitative interview study. European Child & Adolescent Psychiatry, 31, 891–907. 33502596 10.1007/s00787-020-01707-0 PMC9209355

[papt12452-bib-0036] Richards, D. A. , Ekers, D. , McMillan, D. , Taylor, R. S. , Byford, S. , Warren, F. C. , Barrett, B. , Farrand, P. A. , Gilbody, S. , Kuyken, W. , O'Mahen, H. , Watkins, E. R. , Wright, K. A. , Hollon, S. D. , Reed, N. , Rhodes, S. , Fletcher, E. , & Finning, K. (2016). Cost and outcome of behavioural activation versus cognitive behavioural therapy for depression (COBRA): Randomised, controlled, non‐inferiority trial. Lancet, 388, 871–880. 27461440 10.1016/S0140-6736(16)31140-0 PMC5007415

[papt12452-bib-0037] Ruggiero, K. J. , Morris, T. L. , Hopko, D. R. , & Lejuez, C. W. (2007). Application of behavioral activation treatment for depression to an adolescent with a history of child maltreatment. Clinical Case Studies, 6(1), 64–78.

[papt12452-bib-0038] Shenton, N. , Redmond, T. , Kroll, L. , & Parry, S. (2021). Exploring behavioural activation as a treatment for low mood within CAMHS: An IPA study of adolescent experiences. Clinical Child Psychology and Psychiatry, 26, 1–17. 10.1177/13591045211031743 34250833

[papt12452-bib-0039] Shorey, S. , Ng, E. D. , & Wong, C. H. J. (2022). Global prevalence of depression and elevated depressive symptoms among adolescents: A systematic review and meta‐analysis. The British Journal of Clinical Psychology, 61(2), 287–305. 34569066 10.1111/bjc.12333

[papt12452-bib-0040] Skivington, K. , Matthews, L. , Simpson, S. A. , Craig, P. , Baird, J. , Blazeby, J. M. , Boyd, K. A. , Craig, N. , French, D. P. , McIntosh, E. , Petticrew, M. , Rycroft‐Malone, J. , White, M. , & Moore, L. (2021). A new framework for developing and evaluating complex interventions: Update of Medical Research Council guidance. BMJ, 30, 374. 10.1136/bmj.n2061 PMC8482308 34593508

[papt12452-bib-0041] Tiemens, B. , Kloos, M. , Spijker, J. , Ingenhoven, T. , Kampman, M. , & Hendriks, G.‐J. (2019). Lower versus higher frequency of sessions in starting outpatient mental health care and the risk of a chronic course; a naturalistic cohort study. BMC Psychiatry, 19, 228. 31340791 10.1186/s12888-019-2214-4 PMC6657162

[papt12452-bib-0042] Tindall, L. , Mikocka‐Walus, A. , McMillan, D. , Wright, B. , Hewitt, C. , & Gascoyne, S. (2017). Is behavioural activation effective in the treatment of depression in young people? A systematic review and meta‐analysis. Psychology and Psychotherapy, 90(4), 770–796. 28299896 10.1111/papt.12121 PMC5697579

[papt12452-bib-0043] Tindall, L. , Toner, P. , Mikocka‐Walus, A. , & Wright, B. (2021). Perceptions of and opinions on a computerized behavioral activation program for the treatment of depression in young people: Thematic analysis. Journal of Medical Internet Research, 23(4), e19743. 33847594 10.2196/19743 PMC8080144

[papt12452-bib-0044] Wallis, A. , Roeger, L. , Milan, S. , Walmsley, C. , & Allison, S. (2012). Behavioural activation for the treatment of rural adolescents with depression. The Australian Journal of Rural Health, 20, 95–96. 22435770 10.1111/j.1440-1584.2012.01261.x

[papt12452-bib-0045] Watson, R. , Harvey, K. , Pass, L. , McCabe, C. , & Reynolds, S. (2021). A qualitative study exploring adolescents' experience of brief behavioural activation for depression and its impact on the symptom of anhedonia. Psychology and Psychotherapy: Theory, Research and Practice, 94, 266–288. 10.1111/papt.12307 32918843

[papt12452-bib-0046] Weisz, J. R. , Doss, A. J. , & Hawley, K. M. (2005). Youth psychotherapy outcome research: A review and critique of the evidence base. Annual Review of Psychology, 56, 337–363. 10.1146/annurev.psych.55.090902.141449 15709939

[papt12452-bib-0047] World Health Organization . (2014). Adolescents: Health risks and solutions. World Health Organization.

