# Supplementary material for: Barriers and enablers for young people, parents and therapists undertaking behavioural activation for depression: A qualitative evaluation within a randomised controlled trial
Source: Psychol Psychother. 2023 Feb 20;96(2):504–24. doi: 10.1111/papt.12452 (PMC10952148; doi:10.1111/papt.12452)
Supplement: Supplementary file 1 — Appendix S1. [file PAPT-96-504-s001.docx]

**Online Supplementary Materials: Interview Topic Guides**
